# StonPy: a tool to parse and query collections of SBGN maps in a graph database

**DOI:** 10.1093/bioinformatics/btad100

**Published:** 2023-03-10

**Authors:** Adrien Rougny, Irina Balaur, Augustin Luna, Alexander Mazein

**Affiliations:** Biotechnology Research Institute for Drug Discovery, National Institute of Advanced Industrial Science and Technology (AIST), Tokyo 135-0064, Japan; Com. Bio Big Data Open Innovation Lab. (CBBD-OIL), AIST, Tokyo 169-8555, Japan; Luxembourg Centre for Systems Biomedicine (LCSB), University of Luxembourg, 7, avenue des Hauts Fourneaux, Esch-sur-Alzette L-4362, Luxembourg; Department of Systems Biology, Harvard Medical School, Boston, MA, USA; Department of Data Science, Dana-Farber Cancer Institute, Boston, MA, USA; Luxembourg Centre for Systems Biomedicine (LCSB), University of Luxembourg, 7, avenue des Hauts Fourneaux, Esch-sur-Alzette L-4362, Luxembourg

## Abstract

**Summary:**

The systems biology graphical notation (SBGN) has become the *de facto* standard for the graphical representation of molecular maps. Having rapid and easy access to the content of large collections of maps is necessary to perform semantic or graph-based analysis of these resources. To this end, we propose StonPy, a new tool to store and query SBGN maps in a Neo4j graph database. StonPy notably includes a data model that takes into account all three SBGN languages and a completion module to automatically build valid SBGN maps from query results. StonPy is built as a library that can be integrated into other software and offers a command-line interface that allows users to easily perform all operations.

**Availability and implementation:**

StonPy is implemented in Python 3 under a GPLv3 license. Its code and complete documentation are freely available from https://github.com/adrienrougny/stonpy.

**Supplementary information:**

Supplementary data are available at *Bioinformatics* online.

## 1 Introduction

The systems biology graphical notation (SBGN) (Le Novere *et al.*, 2009) is one of the main standards for representing molecular networks graphically. SBGN includes three different languages: process description (PD), for representing reaction networks (Rougny *et al.*, 2019); activity flow (AF), for representing influence graphs (Mi *et al.*, 2015); and entity relationship (ER), for representing rule-based graphs (Sorokin *et al.*, 2015). Having fast and easy access to the content of maps is valuable for semantic and graph-based analysis. For this purpose, Touré *et al.* (2016) previously proposed STON (SBGN to Neo4j), a Java-based software for storing SBGN PD and AF maps into a Neo4j database. Neo4j (https://neo4j.com/) is a freely available labeled property graph-based database that (i) provides R, Java and Python-based APIs, (ii) uses Cypher, a declarative graph query language and (iii) facilitates user query and exploration via a web-based graphical user interface. Information within a Neo4j database is represented by *labeled nodes* (for concepts) and typed edges called *relationships* (for relationships between concepts); more, attributes of concepts and of their inter-relationships can be stored as named values called *properties* (with numeric, Booleans or string values). Graph databases have been proven efficient for storing and querying molecular networks (Fabregat *et al.*, 2018). STON, however, revealed a few drawbacks: first, it does not support the latest versions of Neo4j; second, it is available as standalone software, which makes it difficult to integrate into programmatic pipelines; third, its data model does not support all three SBGN languages (in particular, it does not support ER maps); and finally, features such as state variables and units of information cannot be queried efficiently, and complex queries are needed instead. To solve these issues, we developed StonPy, a new tool to store SBGN maps into a Neo4j database. Specifically, StonPy includes a new comprehensive data model that takes into account all elements of the three SBGN languages in a way they can be easily queried, including annotations. It also offers new capabilities to retrieve (sub-)maps from the database, based on a completion module that automatically builds valid SBGN maps from query results representing parts of maps.

## 2 StonPy data model

**Fig. 1.**
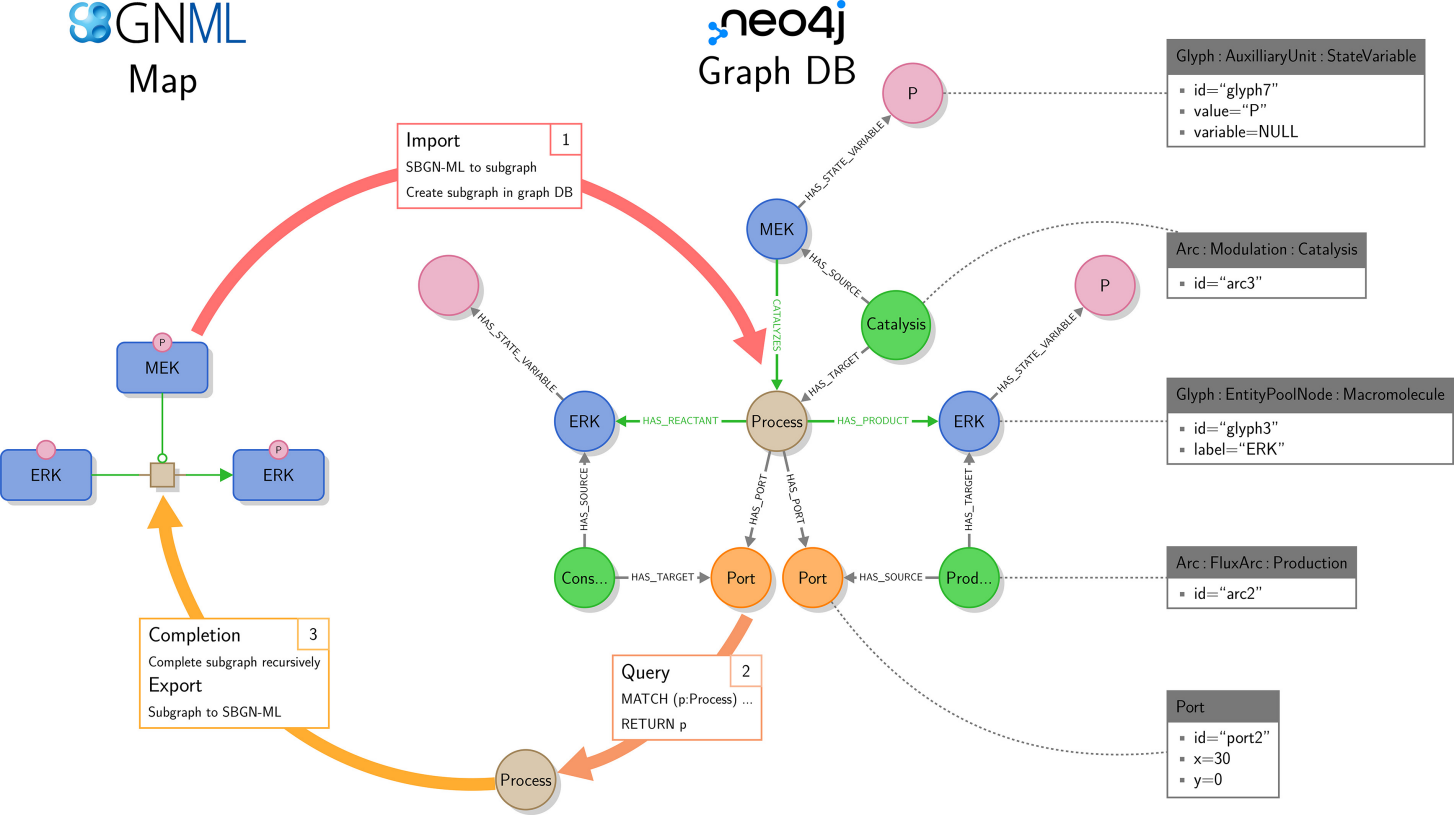
Overview of StonPy’s functionalities. An example of an input SBGN PD map is shown on the left. The corresponding Neo4j graph, built using StonPy, is shown on the right. All SBGN glyphs and arcs are modeled using Neo4j nodes; relationships between SBGN glyphs and sub-glyphs or complex attributes are modeled using Neo4j relationships. SBGN arcs are optionally modeled using additional Neo4j relationships (CATALYZES, HAS_REACTANT, and HAS_PRODUCT relationships) that mimic the structure of the SBGN map and facilitate writing queries on the represented biological concepts. For each Neo4j node, either one of its labels or one of its attributes is shown; for each label, its type is shown. Examples of labels and attributes are shown for some Neo4j nodes in the boxes on the right. Consumption (Cons) and Production (Prod) have been shortened for visualization

The data model of StonPy takes into account all SBGN elements in a way they can be easily queried (see Supplementary Table S1 for a quick comparison with STON’s data model). Each element of an SBGN map is stored as a Neo4j node, and each relationship between two elements (including between an element and one of its sub-elements) is modeled as a Neo4j relationship connecting the nodes modeling the two elements (Fig. 1).

In our data model, SBGN arcs are stored using Neo4j nodes. This structure is required since arcs may contain sub-elements [such as (sub-)glyphs or ports] that are themselves modeled using nodes. It has however two drawbacks: first, it moves our model away from the representation of maps itself, where arcs are links between nodes, and makes it less intuitive; second, it makes it more difficult for the user to write queries focusing on the biological concepts only, without taking into account their specific representation. To solve these issues, each arc is additionally modeled using a graph relationship from the node modeling its source to the one modeling its target (Fig. 1, green relationships).

## 3 StonPy functionalities and command-line tool

The StonPy library allows users to store SBGN-ML (Bergmann *et al.*, 2020) maps into a running Neo4j database, and conversely retrieve them into SBGN-ML (Fig. 1, Import and Export functionalities). StonPy also includes a completion module that allows users to build valid SBGN maps from query results representing parts of maps automatically (Fig. 1, Query and Completion functionalities). This module significantly facilitates the extraction of submaps from the database based on given criteria expressed in a query. The completion algorithm and a complete example are given in Supplementary Information (Supplementary Table S2 and Supplementary Fig. S1). StonPy also includes a command-line tool that allows users to easily perform all operations permitted by the library, and to automatically download SBGN and CellDesigner maps from a number of publicly available map repositories [e.g. the PANTHER database (Mi *et al.*, 2019) or the Atlas of Cancer Signalling Network (Kuperstein *et al.*, 2015)] and store them into a Neo4j database.

## 4 StonPy use cases

StonPy brings new capabilities for storing and analyzing large collections of CellDesigner and SBGN maps using Neo4j and Cypher. It has been successfully tested in several projects, demonstrating its usefulness in real-case applications, and how it can be integrated into workflows for the design and analysis of maps (Mazein *et al.*, 2022). For example, StonPy has been used to build a Neo4j database integrating the maps of the COVID-19 Disease Map resource (Niarakis *et al.*, 2022), and for the extensive analysis and comparison of the maps of the PANTHER database and Atlas of Cancer Signalling Networks resources (Mazein *et al.*, 2021; Rougny *et al.*, 2021).

## Supplementary Material

btad100_Supplementary_Data

## Data Availability

StonPy is implemented in Python 3 under a GPLv3 license. It relies on the libsbgn-python library König (2020) for reading SBGN-ML files and on the py2neo library (https://py2neo.org/) for interfacing with Neo4j. StonPy’s code and a complete documentation are freely available from https://github.com/adrienrougny/stonpy.
