# Supplementary material for: StonPy: a tool to parse and query collections of SBGN maps in a graph database
Source: Bioinformatics. 2023 Mar 10;39(3):btad100. doi: 10.1093/bioinformatics/btad100 (PMC10017094; doi:10.1093/bioinformatics/btad100)
Supplement: btad100_Supplementary_Data [file btad100_supplementary_data.zip › rev_supp_mat.pdf]

StonPy: a tool to parse and query collections of SBGN maps in a graph database
Supplementary Information

January 14, 2023

1 StonPy's vs STON's data model

Table S1 gives a comparison between StonPy's and STON's data models. There are two major differences between the two models: First, arcs are modelled using relationships in STON. As a consequence, STON cannot take into account arcgroups (which are structures that contain arcs) and does not support SBGN ER maps. Because StonPy models arcs using nodes, it can take into account arcgroups and fully supports SBGN ER maps. Second, all subglyphs but subunits are modelled using node properties in STON, which makes difficult to write queries related to subglyphs. On the contrary to STON, they are modelled using nodes in StonPy, and can be easily queried.

**SBGN-ML element**	**STON**	**StonPy**
Glyph	Node	Node + Relationship to owning node
Arc	Relationship between source and target	Node + Relationship to owning node + Relationship between source and target (optional)
Arcgroup	*Not supported*	Node + Relationship to owning node
Map	Property of owned node	Node
Annotation	*Not supported*	Node + Relationship to owning node
Label	Property of owning node	Node + Relationship to owning node
Bbox	Property of owning node	Node + Relationship to owning node
Unit of information of a glyph	Property of owning node (list of strings)	Node + Relationship to owning node
Sate variable of a glyph	Property of owning node (list of strings)	Node + Relationship to owning node
Port of a glyph	Property of owning node	Node + Relationship to owning node
Subunit of a complex	Node + Relationship to owning node	Node + Relationship to owning node
Source/Target of an arc	Property of owning relationship	Relationship to owning node

Table S1: **Stonpy's data model compared to the one of STON.**

2 StonPy's completion module

When the result of a query is a subgraph, it may be completed to form a "complete subgraph" using StonPy's completion module. The "complete subgraph" can then be converted to a valid SBGN map using StonPy's conversion module. To form the "complete subgraph", the completion algorithm runs through all relationships and nodes of the input subgraph and completes them (i.e., adds nodes and relationships to the subgraph) following the completion rules described below. Completion may be recursive: a completion rule may add a node or a relationship to the list of nodes and relationships to be completed.

2.1 Relationships

A relationship is completed by its source and target nodes, which are themselves completed. A relationship modelling an arc (such as CATALYZES, INHIBITS, PRODUCES, etc.) is also completed with the graph node modelling the same arc, which is itself also completed.

2.2 Nodes

Nodes are completed following the rules given in Table S2. A completion rule is applied to a given node only if that node includes the label given in the "Node" column. If this is the case, the Neo4j database is queried for all relationships having the type given in the "Relationship type" column and having the node as its source or target, depending on the value in the "Node role" column. The node is then completed with the resulting relationships, which are themselves not completed. The node is also completed with the "other node" of each relationship (i.e., the target node if the initial node to be completed is the source of the relationship, and the source node otherwise), which is itself completed if the value of the "Recursive" column is "Yes".

For example, the first rule states that any node that models a glyph (i.e., that includes the "Glyph" label) should be completed with all relationships in the Neo4j graph whose type is "HAS_LABEL" and whose source is the node. Furthermore, the target of such relationships should also be completed.

2.3 Example

We consider a Neo4j database storing the map given in Figure 1. We make the following query to the database: `MATCH (p:StoichiometricProcess) RETURN p`. The result of this query is a unique Neo4j node `(p)` that models the unique process glyph of the map. This Neo4j node cannot be converted to a valid SBGN-ML map as many other Neo4j nodes and relationships are lacking (e.g., the nodes and relationships modelling the reactants and products of the process). Hence we use StonPy's completion module to complete this Neo4j node. The algorithm performs completion rules in the order given in Figure S1A to output the "complete subgraph" given in Figure S1B. This "complete subgraph" can then be converted to a valid SBGN-ML map using StonPy's conversion module.

Rule number	Node	Relationship type	Node role	Recursive
1	Glyph	HAS_LABEL	Source	Yes
2	Glyph	HAS_BBOX	Source	No
3	Glyph	IS_IN_COMPARTMENT	Source	Yes
4	Glyph	HAS_STATE_VARIABLE	Source	Yes
5	Glyph	HAS_UNIT_OF_INFORMATION	Source	Yes
6	Glyph	HAS_SUBUNIT	Source	Yes
7	Glyph	HAS_OUTCOME	Source	Yes
8	Glyph	HAS_TERMINAL	Source	Yes
9	Glyph	HAS_EXISTENCE	Source	Yes
10	Glyph	HAS_LOCATION	Source	Yes
11	Glyph (if not AuxilliaryUnit)	HAS_GLYPH	Target	No
12	StoichiometricProcess	HAS_PORT	Source	No
13	StoichiometricProcess (if complete_process_modulations is True)	HAS_TARGET	Target	Yes
14	LogicalOperator	HAS_PORT	Source	Yes
15	EquivalenceOperator	HAS_PORT	Source	Yes
16	AuxilliaryUnit	HAS_GLYPH	Target	Yes
17	Port	HAS_PORT	Target	Yes
18	Port	HAS_SOURCE	Target	Yes
19	Port	HAS_TARGET	Target	Yes
20	Bbox	HAS_BBOX	Target	Yes
21	Start	HAS_START	Target	Yes
22	Next	HAS_NEXT	Target	Yes
23	End	HAS_END	Target	Yes
24	Label	HAS_LABEL	Target	Yes
25	Arc	HAS_PORT	Source	No
26	Arc	HAS_OUTCOME	Source	Yes
27	Arc	HAS_ARC	Target	No
28	Arc	HAS_CARDINALITY	Source	Yes
29	Arc	HAS_SOURCE	Source	Yes
30	Arc	HAS_TARGET	Source	Yes
31	Arc	HAS_START	Source	No
32	Arc	HAS_END	Source	No
33	Arc	HAS_NEXT	Source	No
34	Arcgroup	HAS_ARC	Source	Yes
35	Arcgroup	HAS_ARCGROUP	Target	No
36	Map	HAS_GLYPH	Source	Yes
37	Map	HAS_ARC	Source	Yes
38	Map	HAS_ARCGROUP	Source	Yes

Table S2: **Completion rules for nodes executed by the completion algorithm.**

A

- (S...Process)
  - ↳ R12: (S...Process)-[HAS_PORT]→(Port)
    - ↳ R18: (Port)←[HAS_SOURCE]-(Prod...)
      - ↳ R30: (Prod...)-[HAS_TARGET]→(ERK)
        - ↳ R4: (ERK)-[HAS_STATE_VARIABLE]→(P)
  - ↳ R12: (S...Process)-[HAS_PORT]→(Port)
    - ↳ R19: (Port)←[HAS_TARGET]-(Cons...)
      - ↳ R29: (Cons...)-[HAS_SOURCE]→(ERK)
        - ↳ R4: (ERK)-[HAS_STATE_VARIABLE]→()
  - ↳ R13: (S...Process)←[HAS_TARGET]-(Catalysis)
    - ↳ R29: (Catalysis)-[HAS_SOURCE]→(MEK)
      - ↳ R4: (MEK)-[HAS_STATE_VARIABLE]→(P)

B

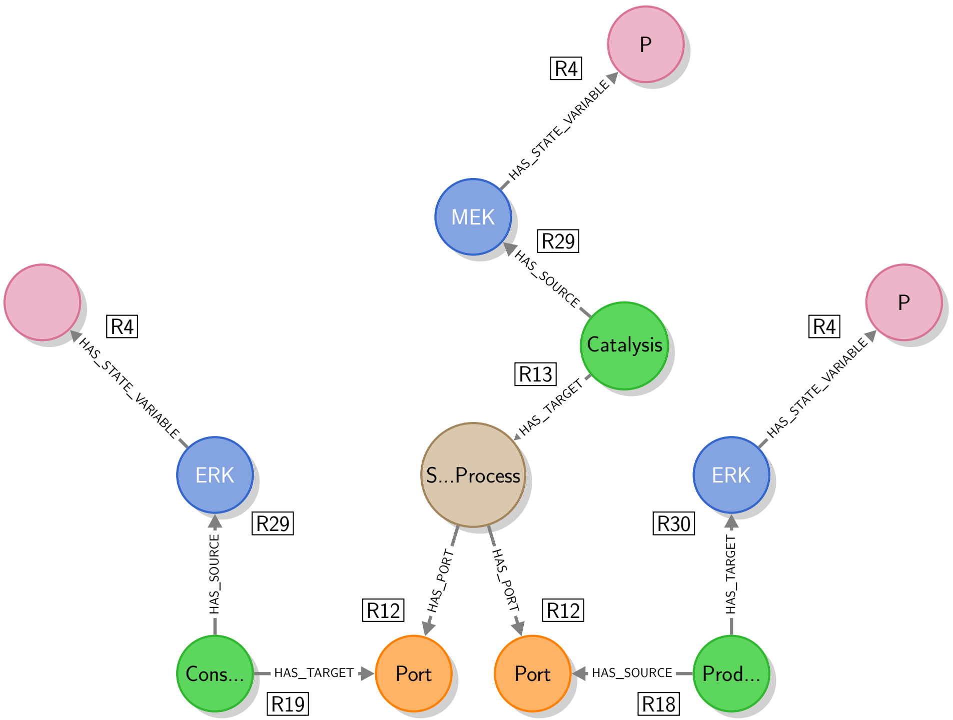

Figure S1: **Completion of the result of a query.** We consider the query `MATCH (p:StoichiometricProcess) RETURN p` on the database formed of the map of Figure 1. This query returns only one node, which models the only process of the map. **A** Rules that are performed by StonPy's completion algorithm. Rule numbers are those of Table S2. **B** The output "complete subgraph". This subgraph can then be converted to a valid SBGN map using StonPy's conversion module. For the sake of readability some of the rules that are performed are omitted (e.g., rule 2 is performed for each Neo4j node modelling a glyph but is not shown).
