# Supplementary material for: StonPy: a tool to parse and query collections of SBGN maps in a graph database
Source: Bioinformatics. 2023 Mar 10;39(3):btad100. doi: 10.1093/bioinformatics/btad100 (PMC10017094; doi:10.1093/bioinformatics/btad100)
Supplement: btad100_Supplementary_Data [file response_to_reviewers.pdf › response_to_reviewers.pdf]

We thank the reviewers for their thorough review and useful comments. Our response to these comments and the various improvements made to the manuscript, the software, and the documentation can be found below, in blue.

The main changes brought to the manuscript are i) the replacement of all occurrences of the name "*stonpy*" by the new name "StonPy" and ii) the addition of a mention of real world use cases of the StonPy software, that demonstrate how StonPy can be useful for analyses of large collections of maps in systems biology.

The main changes brought to StonPy's documentation are i) the addition of code example in the documentation of each class and function of the core module, ii) a new jupyter notebook that gathers these pieces of code into a unique executable tutorial document and iii) an extended documentation for StonPy's command line interface.

Reviewer: 1

Comments to the Author

Major:

The authors promise a supplementary file with figures / tables, those are not available with the article. This should be remedied.

While we provided it for the initial submission and checked its accessibility in the online system, we are sorry the supplementary material could not be found by the reviewer. We have resubmitted the supplementary information with this revision. The file is also available at https://github.com/adrienrougny/stonpy/blob/master/docs/supp_mat.pdf.

General:

As always documentation of the python package can (and should be improved):

- The stonpy module, should feature a __version__ field, to quickly verify the correct version

We added a __version__ attribute to the StonPy package.

- The 0.1 version of the package should be removed from pypi, as it will install silently python versions < 3.10, and not contain the described features.

We thank the reviewer for noticing this outdated version. We removed version 0.1 from pypi, which now only features versions 0.1.1 and 0.2.1 (latest).

- After installation the stonpy module is not working, unless `python-magic-bin` is installed. This should happen automatically, or pointed out in the documentation.

We indeed missed this required dependency for the Windows system. We added it to the package setup configuration so that it is installed automatically on this system.

- The command line switches, and a worked example using them would be nice to see available at the website. that way users don't have to look through the source code themselves. For example, how should the repositories be used?

We built a new complete documentation for StonPy's CLI, available at https://stonpy.readthedocs.io/en/latest/cli.html. This documentation includes an example that demonstrates how to use the different subcommands, including the use of repositories.

- While an example file is provided in the example folder, it would be nice to have it accessible from the documentation, together with the output it produces. That would make it easier for users to get started.

We built a new jupyter notebook that gathers the snippets of code of the documentation into a unique executable tutorial document. This new notebook is available at https://github.com/adrienrougny/stonpy/blob/master/notebooks/tutorial.ipynb.

Reviewer: 2

Comments to the Author

The authors present a new Python-based tool that parses and writes out files in SBGNML format with a Neo4j graph database backend in this work. It provides functions to query the database. A supplementary document explains some key features in more detail.

Neo4j graph databases are becoming increasingly popular, particularly in analyzing biological networks. It is remarkable that a general solution for storing and querying SBGN graphs is now available.

However, the manuscript and the software need some extra effort for publication.

Major points:

* Please demonstrate the software with a realistic but small use case example. There is still space on the back of the two-page paper where another subfigure could fit that indicates how the software can solve a real problem.

We added the following section regarding use cases:

"4 StonPy use cases

StonPy brings new capabilities for storing and analyzing large collections of CellDesigner and SBGN maps using Neo4j and Cypher. It has been successfully tested in several projects, demonstrating its usefulness in real case applications, and how it can be integrated into workflows for the design and analysis of maps [6]. In particular, StonPy has been used to build a Neo4j database integrating the maps of the COVID-19 Disease Map resource [10], and for the extensive analysis and comparison of the maps of the PANTHER database and Atlas of Cancer Signalling Networks resources [11, 7]."

* The read-the-docs page is minimal and needs more information for users. Please extend the documentation.

The documentation was extended with i) a code example of each class and function of the core module, ii) a new jupyter notebook that gathers these pieces of code into a unique executable tutorial document and iii) an extended documentation for StonPy's command line interface. The notebook is available at https://github.com/adrienrougny/stonpy/blob/master/notebooks/tutorial.ipynb, while the new documentation for the command line interface is available at https://stonpy.readthedocs.io/en/latest/cli.htm.

Minor points:

* Was the SBGNML format forgotten to be cited?

We added the citation at the first mention of SBGN-ML.

* There are a few typos in both the manuscript and the supplement that need correction. This reviewer cannot list all of them, but here are a few hints:

We thank the reviewer for pointing all these typos out. We took them all into account both in the article and in the supplementary material.

Concerning the first point below, we changed the name of the tool from *stonpy* to StonPy in the article, the supplementary material, and in the documentation.

1) The article:

- Avoid starting sentences, the title, or several headlines with a lowercase letter. Find another way to change the text so that "stonepy" either begins with an upper-case "S" or is written in a different position. Sentences beginning with a lowercase word are irritating.

- Introduction, the enumeration is wrong (iii twice).

- Boolean should be written with an upper case because it goes back to a person's name.

- cannot is one word

- “in order to” can often be shortened to “to.”

- a line should not start with an opening parenthesis

- In the references, many titles are all lowercase (even “sbgn,” etc.)

- The font inside of Fig 1 is tiny and can certainly not be read in the article’s print version as is.

2) The supplement:

- which without h at the end

- commas are forgotten after “i.e.” and “e.g.” (many times)
